# The reciprocal causal relationship between frailty and physical activity among Korean older adults by age groups

**DOI:** 10.1093/geroni/igaa057.3345

**Published:** 2020-12-16

**Authors:** Hae Sagong, Ju Young Yoon

**Affiliations:** Seoul National University, Seoul, Republic of Korea

## Abstract

Among associated factors to frailty, physical activity is a highly recommended intervention that prevents the risk factors of the frailty. However, most of the older adults are lack of sufficient physical activity to obtain health-related benefits. The purpose of this study is to investigate the reciprocal relationship between frailty and physical activity among Korean older adults by age groups of middle-old (70-79) and oldest-old (more than 80) between two years using cross-lagged panel analysis. This study is a secondary data analysis of the Korean Frailty and Aging Cohort Study (KFACS) and a total of 1,092 participants were included. Frailty was measured by the FRAIL scale and physical activity was measured by the International Physical Activity Questionnaire (IPAQ). As for the result, in the middle-old group, frailty and high PA had significant reciprocal causal relationships while moderate PA with frailty had no significant relationship reference to low PA. In short, frailty was associated with less high PA, and high PA predicts less frailty after two years. In the oldest-old group, surprisingly, there was no reciprocal causal relationship between frailty and any level of PA reference to low PA which means PA has no effects on frailty and vice versa. This can be explained by the ceiling effect or overestimation of the physical activity. Therefore, further studies on the relationship between frailty and physical activity of the oldest-old population are needed. Also, specific physical activity guidelines and effective measurement of physical activity for older adults by age segment should be developed.

